# Complete chloroplast genome of *Stephania tetrandra* (Menispermaceae) from Zhejiang Province: insights into molecular structures, comparative genome analysis, mutational hotspots and phylogenetic relationships

**DOI:** 10.1186/s12864-021-08193-x

**Published:** 2021-12-06

**Authors:** Shujie Dong, Zhiqi Ying, Shuisheng Yu, Qirui Wang, Guanghui Liao, Yuqing Ge, Rubin Cheng

**Affiliations:** 1 School of Pharmaceutical Sciences, Zhejiang Chinese Medical University, 548 Binwen Road, Hangzhou, Zhejiang Province People’s Republic of China; 2 The Administration Bureau of Zhejiang Jiulongshan National Nature Reserve, Suichang, Zhejiang Province People’s Republic of China; 3 The First Affiliated Hospital of Zhejiang Chinese Medical University, 54 Youdian Road, Hangzhou, Zhejiang Province People’s Republic of China

**Keywords:** *Stephania tetrandra*, Chloroplast genome, Phylogenetic relationship, Comparative analysis, Mutational hotspots

## Abstract

**Background:**

The *Stephania tetrandra* S. Moore (*S. tetrandra*) is a medicinal plant belonging to the family Menispermaceae that has high medicinal value and is well worth doing further exploration. The wild resources of *S. tetrandra* were widely distributed in tropical and subtropical regions of China, generating potential genetic diversity and unique population structures. The geographical origin of *S. tetrandra* is an important factor influencing its quality and price in the market. In addition, the species relationship within *Stephania* genus still remains uncertain due to high morphological similarity and low support values of molecular analysis approach. The complete chloroplast (cp) genome data has become a promising strategy to determine geographical origin and understand species evolution for closely related plant species. Herein, we sequenced the complete cp genome of *S. tetrandra* from Zhejiang Province and conducted a comparative analysis within *Stephania* plants to reveal the structural variations, informative markers and phylogenetic relationship of *Stephania* species.

**Results:**

The cp genome of *S. tetrandra* voucher ZJ was 157,725 bp, consisting of a large single copy region (89,468 bp), a small single copy region (19,685 bp) and a pair of inverted repeat regions (24,286 bp each). A total of 134 genes were identified in the cp genome of *S. tetrandra*, including 87 protein-coding genes, 8 rRNA genes, 37 tRNA genes and 2 pseudogene copies (*ycf1* and *rps19*). The gene order and GC content were highly consistent in the *Stephania* species according to the comparative analysis results, with the highest RSCU value in arginine (1.79) and lowest RSCU value in serine of *S. tetrandra*, respectively. A total of 90 SSRs have been identified in the cp genome of *S. tetrandra*, where repeats that consisting of A or T bases were much higher than that of G or C bases. In addition, 92 potential RNA editing sites were identified in 25 protein-coding genes, with the most predicted RNA editing sites in *ndhB* gene. The variations on length and expansion extent to the junction of *ycf1* gene were observed between *S. tetrandra* vouchers from different regions, indicating potential markers for further geographical origin discrimination. Moreover, the values of transition to transversion ratio (Ts/Tv) in the *Stephania* species were significantly higher than 1 using *Pericampylus glaucus* as reference. Comparative analysis of the *Stephania* cp genomes revealed 5 highly variable regions, including 3 intergenic regions (*trnH-psbA*, *trnD-trnY*, *trnP*) and two protein coding genes (*rps16* and *ndhA*). The identified mutational hotspots of *Stephania* plants exhibited multiple SNP sites and Gaps, as well as different Ka/Ks ratio values. In addition, five pairs of specific primers targeting the divergence regions were accordingly designed, which could be utilized as potential molecular markers for species identification, population genetic and phylogenetic analysis in *Stephania* species. Phylogenetic tree analysis based on the conserved chloroplast protein coding genes indicated a sister relationship between *S. tetrandra* and the monophyletic group of *S. japonica* and *S. kwangsiensis* with high support values, suggesting a close genetic relationship within *Stephania* plants. However, two *S. tetrandra* vouches from different regions failed to cluster into one clade, confirming the occurrences of genetic diversities and requiring further investigation for geographical tracing strategy.

**Conclusions:**

Overall, we provided comprehensive and detailed information on the complete chloroplast genome and identified nucleotide diversity hotspots of *Stephania* species. The obtained genetic resource of *S. tetrandra* from Zhejiang Province would facilitate future studies in DNA barcode, species discrimination, the intraspecific and interspecific variability and the phylogenetic relationships of *Stephania* plants.

**Supplementary Information:**

The online version contains supplementary material available at 10.1186/s12864-021-08193-x.

## Background

The *Stephania tetrandra* S. Moore (*S. tetrandra*) is a kind of *Stephania* plants belonging to the tribe Menispermea of family Menispermaceae. It is a perennial herb that grows in the bush near the village, wilderness and roadside, and is widely distributed in the southern part of China [1]. The dried root of *S. tetrandra* is used as the traditional Chinese medicine Fangji in China with various pharmacological effects including diuretic, anti-inflammatory and an antirheumatic treatment [2]. Up to data, a total of 67 alkaloids and several other active compounds have been isolated and characterized from the roots and aerial parts of *S. tetrandra* [2]. The typical bisbenzylisoquinoline alkaloidhas tetrandrine derived from *S. tetrandra* have shown multiple biological activities, like anti-tumor, anti-inflammatory, prevention and treatment of various fibrotic diseases [3–5]. Tetrandrine was also reported to inhibit migration and invasion of human nasopharyngeal carcinoma dependent of MAPK and RhoA signaling pathways, suggesting its potential development in the application of anticancer agents for nasopharyngeal carcinoma therapy [6]. In addition, Fangji Huangqi Decoction was reported to protect the kidney directly and reduce the level of uric acid indirectly [7]. Therefore, the *S. tetrandra* has high medicinal value and is worthwhile to do further exploration.

According to the Chinese Pharmacopoeia, *S. tetrandra* is the only authentic source of Fangji [8]. However, due to the similarity in name and morphology, *S. tetrandra* is often mistakenly substituted and adulterated with roots from other plants, which contained different chemical compositions and therapeutic effects, such as *Aristolochia fangchi* [9]. In addition, the closely related Menispermaceae species of *Cocculus orbiculatus*, *Menispermum dauricum* and *Sinomenium acutum* also provided potential counterfeits for *S. tetrandra* in the market. The problem of wrong substitutions brings potential safety hazard and might leads damage to health. In addition, the classification of *Stephania* species has been conducted for many years using different methods. The traditional classification methods divided genus *Stephania* into three subgenera based on the comparison of appearance characters of plants [10]. With the development of molecular biology and DNA sequencing technology, molecular systematics research methods have also been used for taxonomic studies of the genus *Stephania* [11]*.* The DNA markers *ITS* and *trnL-F* have been applied to construct the phylogenetic tree of *Stephania* species. However, the analysis results gave controversial phylogenetic relationship among the three subgenera of the genus *Stephania* [11, 12]. Compared with the nuclear gene and partial chloroplast genes, the complete chloroplast genome with a large set of genes is generally considered as a useful tool for phylogenetic analysis and further development on species identification and restoration strategies [13]. The chloroplast genome of plants contains a lot of molecular information that is a good resource for plant systematics, population genomics and phylogenetic studies [14]. Thus, it is important to sequence the complete chloroplast genome and develop highly variable and informative markers to study the reticulate evolution history of the genus *Stephania* and to identify *S. tetrandra* and other adulterants accurately to ensure the clinical safety of *S. tetrandra*.

The chloroplast, which performs photosynthesis, is an essential organelle in plants and is generally non-recombinant and uniparentally inherited. In recent years, chloroplast genomic information has been widely used for developing molecular markers to classify medicinal plants and used for evolutionary studies [15, 16]. In most angiosperms, chloroplast genome is generally double stranded and circular [17]. It consists of a small single copy region (SSC), a large single copy region (LSC) and a pair of inverted repeat regions (IR) which separate SSC and LSC [18]. On average, the size of chloroplast genome varies from 115 kb to 165 kb [17]. There are many studies have reported that the chloroplast genome is expected to serve as a super-barcode to distinguish closely related species [19]. Comparative and phylogenetic analysis of six *Ligularia* species observed a close relationship between *L. fischeri* and *L. jaluensis* and the whole cp genome was expected to become a super-barcode to identify *Ligularia* species [20]*.* The *ycf1*b region from the complete chloroplast genome of *Pterocarpus*, was identified as a potential molecular marker for species identification of *Pterocarpus* wood [21]. The complete chloroplast genome sequence of *S. tetrandra* from Jiangxi Province has been determined previously [22]. However, the chloroplast genome information of *S. tetrandra* from Zhejiang Province has not been characterized and a comprehensive analysis of the chloroplast structures of *Stephania* species is still lacking. The aims of this study were to (i) conduct comparative research on the *Stephania* chloroplast genome, generating information on basic genome structure, codon usage bias, repetitive structure characteristics, and RNA editing sites; (ii) identify hotspot regions and develop potential DNA markers through high nucleotide diversity, and (iii) reconstruct the phylogenetic relationships of Menispermaceae species and determine the taxonomic status of *Stephania* based on the conserved chloroplast protein-coding genes data. Our results will be important gene basic data for the marker development, species discrimination, and the inference of phylogenetic relationships for family Menispermaceae based on these comprehensive analyses of chloroplast genomes.

## Results

### Chloroplast genome features of *Stephania tetrandra*

**Fig. 1:**
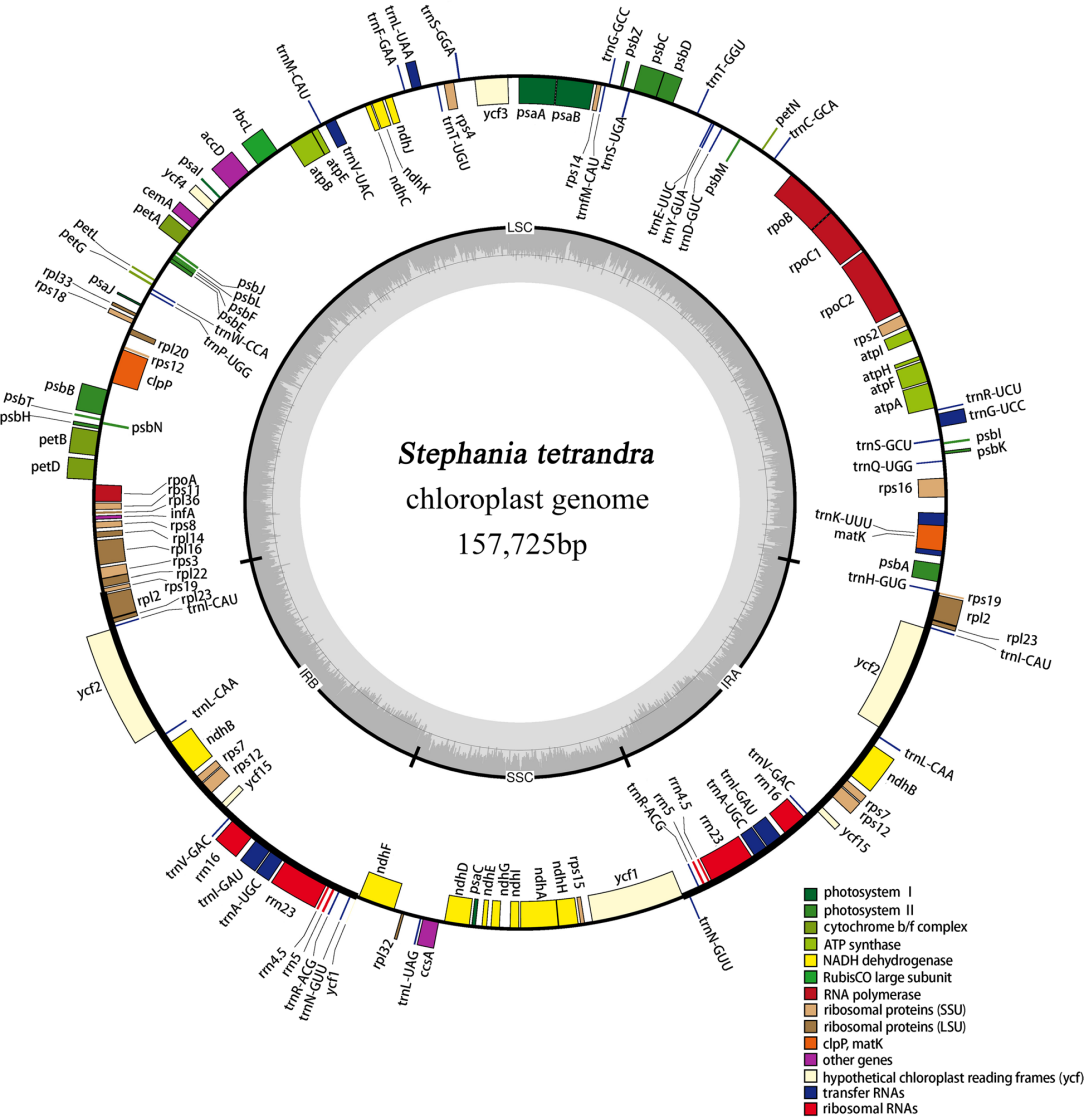
Circular chloroplast (CP) genome map of *Stephania tetrandra*. Genes drawn outside the circle are transcribed anti-clockwise, while those inside the circle are transcribed clockwise. Large single copy (LSC) region, inverted repeat (IRA, IRB) regions and small single copy (SSC) region are shown in the figure. The darker gray in the inner circle corresponds GC content whereas the lighter gray corresponds AT content. Different colors of genes represent their different functions

In total, 21,016,486 Illumina reads and 3,085,951,100 bases were obtained for *Stephania tetrandra* voucher ZJ by Illumina Hiseq 2500 platform. After cutting, selecting and assembling reads, the complete cp genome of *S. tetrandra* with a size of 157,725 bp was generated. It was a circular structure conserved 4 regions: a small single copy region (SSC; 19,685 bp), a large single copy region (LSC; 89,468 bp) and a pair of inverted repeat regions (IR; 24,286 bp of each) which separated SSC and LSC (Fig. 1). As shown in Table 1, the total number of genes annotated in the cp genome of *S. tetrandra* was 134, including 87 protein-coding genes, 8 rRNA genes, 37 tRNA genes and 2 pseudogene copies (*ycf1* and *rps19*). Among the 134 genes, 18 genes were duplicated in IR region and 23 genes contained introns (Table 1). In addition, 11 protein-coding genes (*rps16, petB, petD, atpF, ndhB (× 2), ndhA, rpoC1, rpl16* and *rpl2 (× 2)*) and 8 tRNA genes *(trnK-UUU, trnG-UCC, trnL-UAA, trnV-UAC, trnI-GAU (× 2)* and *trnA-UGC (× 2)*) contained only one intron, while 4 protein-coding genes (*rps12(× 2), clpP* and *ycf3)* had two introns.

**Table 1:** List of genes annotated in the chloroplast genomes of *Staphania tetrandra* voucher ZJ

No.	Group of genes	Gene names	Amount
1	Photosystem I	*psaJ, psaC, psaB, psaA, psaI*	5
2	Photosystem II	*psbE, psbB, psbT, psbN, psbH, psbJ, psbL, psbF, psbA, psbK, psbI, psbM, psbD, psbZ*	14
3	Cytochrome b/f complex	petL, petG, petB*, petD*, petA, petN	6
4	ATP synthase	atpE, atpB, atpA, atpF*, atpH, atpI	6
5	NADH dehydrogense	ndhB*(× 2), ndhF, ndhD, ndhE, ndhJ, ndhK, ndhC, ndhA*, ndhG, ndhI, ndhH	12
6	RubisCO large subunit	*rbcL*	1
7	RNA polymerase	rpoA, rpoC2, rpoC1*, rpoB,	4
8	Ribosomal proteins (SSU)	rps11, rps8, rps3, rps19, rps7, rps14, rps4, rps18, rps12**(×2), rps16*, rps2, rps15	13
9	Ribosomal proteins (LSU)	rpl33, rpl20, rpl36, rpl14, rpl16*, rpl22, rpl2*(×2), rpl23(× 2), rpl32, rpl33, rpl20	13
10	Other genes	clpP**, infA, ccsA, accD, cemA, matK	6
11	Proteins of unknown function	ycf2(×2), ycf3**, ycf4, ycf1, ycf15(× 2)	7
12	Transfer RNAs	*37 tRNAs (8 contain an intron,7 in the IRs)*	37
13	Ribosomal RNAs	*rrn4.5(×2), rrn5(× 2), rrn16(× 2), rrn23(× 2)*	8
14	pseudo genes	*ycf1, rps19*	2

One or two asterisks after a gene indicate that the gene contains one or two introns, respectively

The complete cp genomes of three *Stephania* species showed similarity in terms of the gene content, total length of complete cp genome and composition of GC content. The overall GC contents in the cp genomes of *S. tetrandra* and other *Stephania* species were calculated using MEGA 7.0. The total GC contents of the cp genomes were 38.18, 38.23 and 38.44% in *S. tetrandra*, *S. japonica* and *S. kwangsiensis,* respectively (Table 2). The GC content varied significantly in different regions of the cp genome. IR regions had the highest GC content in all the three cp genomes, followed by LSC region and SSC region. In addition, *ycf1* and *rps19* pseudogenes could be found in all of these three plants. Among their protein-coding genes, the initiation codons of *psbC, rps19, rpl2* and *ndhD* genes were different from the standard initiation codon ATG. The *psbC* and *rps19* genes in the three plants started with GTG, while *rpl2* and *ndhD* in *S. tetrandra*, *S. japonica* started with ACG but in *S. kwangsiensis* started with ATA and GTG respectively. Similar results of *rps19* started with GTG also reported in other Menispermaceae plants [23, 24]. Interestingly, the cp genome of *S. tetrandra* contains two *ycf15* genes in the IR regions, which exhibited the most difference between *S. tetrandra* and the other two *Stephania* species.

**Table 2:** Statistics on the basic features of the chloroplast genomes of the three *Stephania* species

Characteristics	*Stephania tetrandra*	*Stephania japonica*	*Stephania kwangsiensis*
Accession number	MT849286	KU204903.1	MN654112.1
Total length (bp)	157,725	157,719	156,624
LSC length (bp)	89,468	88,693	87,759
SSC length (bp)	19,685	20,346	20,169
IR length (bp)	48,572	48,680	48,696
Total Number of Genes	134	132	132
Coding Genes	87	85	85
rRNA Genes	8	8	8
tRNA Genes	37	37	37
Pesudo genes	2	2	2
GC content			
Total (%)	38.18	38.23	38.44
LSC (%)	36.31	36.42	36.66
SSC (%)	33.00	32.94	33.40
IR (%)	43.73	43.75	43.76

*LSC* Large single copy, *SSC* Small single copy, *IR* Inverted repeat, *GC* Guanine and Cytosine

### Analysis of codon usage bias

**Fig. 2:**
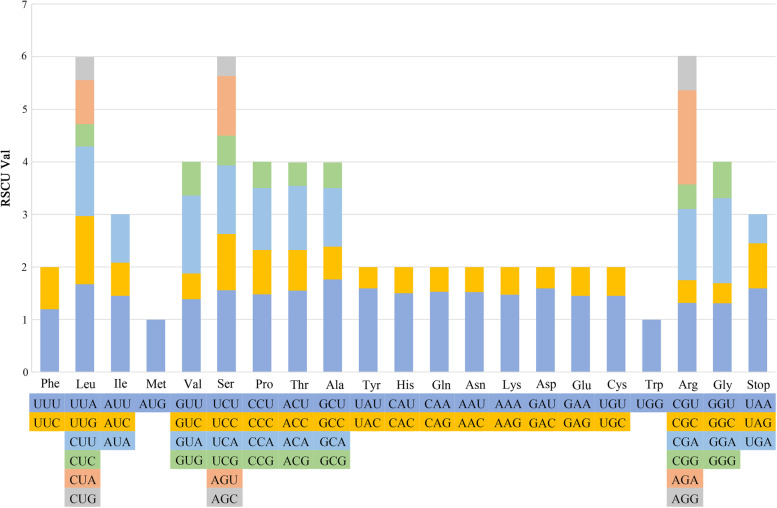
Codon content and RSCU value of the 20 amino acid and stop codons in all protein-coding genes of *Stephania tetrandra* chloroplast genome. The color of the histogram corresponds to the color of codons

In the cp genome of *S. tetrandra*, it contained 78,750 bp of coding sequences consisting of 26,250 codons. Leucine was the most abundant amino acid in the cp genome of *S. tetrandra*, whereas cysteine was the least (Table 3). Regardless of stop codons, the most used codon was AUU, encoding isoleucine (Ile), with a number of 1065, while the least used codon was UGC, encoding cysteine (Cys), with a number of 84 in *S. tetrandra*. Relative synonymous codon usage (RSCU) reflected the ratio of the frequency of usage of a codon to the expected frequency [25]. Most of the amino acid have codon preferences in the cp genome of *S. tetrandra*, however, methionine (AUG) and tryptophan (UGG) were encoded by only one codon and exhibited no codon bias (Fig. 2). AGA (1.79) in arginine showed the highest RSCU value, and the lowest was AGC (0.37) in serine. Comparative analysis indicated the coded amino acids used in *S. japonica* and *S. kwangsiensis* were identical with that of *S. tetrandra*, with the number of codons of 26,005 and 26,019 in *S. japonica* and *S. kwangsiensis*, respectively. Moreover, similar trends on codon usage preference were observed in the three *Stephaniae* plants (Table 3).

**Table 3:** Codon usage and codon-anticodon recognition patterns of three *Stephania* plants

Codon	tRNA	Numbers and RSCU		
		*S. tetrandra*.	*S. japonica*	*S. kwangsiensis*
UUU(F)		870/1.20	882/1.23	889/1.22
UUC(F)	trnF-GAA	576/0.80	557/0.77	568/0.78
UUA(L)		749/1.67	768/1.72	757/1.69
UUG(L)	trnL-CAA	581/1.30	556/1.24	567/1.27
CUU(L)		594/1.32	601/1.35	590/1.32
CUC(L)		193/0.43	185/0.41	189/0.42
CUA(L)		374/0.84	368/0.82	371/0.83
CUG(L)		199/0.44	203/0.45	207/0.46
AUU(I)		1065/1.45	1057/1.45	1056/1.46
AUC(I)	trnI-GAU	464/0.63	447/0.61	460/0.63
AUA(I)		674/0.92	677/0.93	659/0.91
AUG(M)	trnM-CAU	625/1.00	613/1.00	613/1.00
GUU(V)		506/1.39	510/1.41	506/1.39
GUC(V)		179/0.49	175/0.48	181/0.50
GUA(V)		537/1.48	534/1.47	535/1.46
GUG(V)		233/0.64	231/0.64	239/0.65
UCU(S)		540/1.56	526/1.56	524/1.55
UCC(S)	trnS-GGA	371/1.07	362/1.07	370/1.09
UCA(S)	trnS-UGA	451/1.31	432/1.28	428/1.26
UCG(S)		199/0.57	191/0.57	196/0.58
CCU(P)		417/1.48	409/1.46	397/1.42
CCC(P)		236/0.84	234/0.84	253/0.90
CCA(P)	trnP-UGG	331/1.17	318/1.14	321/1.15
CCG(P)		142/0.50	156/0.56	150/0.54
ACU(T)		531/1.55	518/1.54	517/1.54
ACC(T)	trnT-GGU	264/0.77	264/0.78	259/0.77
ACA(T)	trnT-UGU	417/1.22	409/1.21	405/1.21
ACG(T)		155/0.45	158/0.47	159/0.47
GCU(A)		623/1.77	606/1.74	601/1.72
GCC(A)		223/0.63	222/0.64	223/0.64
GCA(A)		392/1.11	376/1.08	386/1.11
GCG(A)		174/0.49	191/0.55	186/0.53
UAU(Y)		747/1.59	749/1.57	731/1.57
UAC(Y)	trnY-GUA	192/0.41	205/0.43	201/0.43
UAA(*)		46/1.59	41/1.45	43/1.52
UAG(*)		25/0.86	26/0.92	26/0.92
CAU(H)		490/1.50	475/1.50	468/1.49
CAC(H)	trnH-GUG	163/0.50	158/0.50	162/0.51
CAA(Q)	trnQ-UUG	731/1.53	718/1.53	724/1.52
CAG(Q)		225/0.47	223/0.47	226/0.48
AAU(N)		961/1.52	940/1.51	948/1.51
AAC(N)		301/0.48	307/0.49	305/0.49
AAA(K)		994/1.47	992/1.46	976/1.46
AAG(K)		362/0.53	366/0.54	364/0.54
GAU(D)		872/1.59	856/1.58	859/1.58
GAC(D)	trnD-GUC	227/0.41	226/0.42	229/0.42
GAA(E)	trnE-UUC	958/1.45	953/1.46	953/1.45
GAG(E)		361/0.55	351/0.54	358/0.55
UGU(C)	trnC-GCA	221/1.45	223/1.46	224/1.49
UGC(C)		84/0.55	82/0.54	77/0.51
UGA(*)		16/0.55	18/0.64	16/0.56
UGG(W)	trnW-CCA	470/1.00	465/1.00	465/1.00
CGU(R)	trnR-ACG	352/1.32	352/1.33	358/1.35
CGC(R)		115/0.43	112/0.42	111/0.42
CGA(R)		360/1.35	343/1.30	352/1.32
CGG(R)		125/0.47	132/0.50	124/0.47
AGU(S)		390/1.13	386/1.14	384/1.13
AGC(S)	trnS-GCU	127/0.37	130/0.38	132/0.39
AGA(R)	trnR-UCU	477.1.79	468/1.77	472/1.77
AGG(R)		174/0.64	176/0.67	179/0.67
GGU(G)		588/1.31	584/1.30	583/1.30
GGC(G)	trnG-GCC	168/0.38	176/0.39	171/0.38
GGA(G)		723/1.62	712/1.59	713/1.59
GGG(G)		311/0.69	324/0.72	323/0.72

### RNA editing sites

**Fig. 3:**
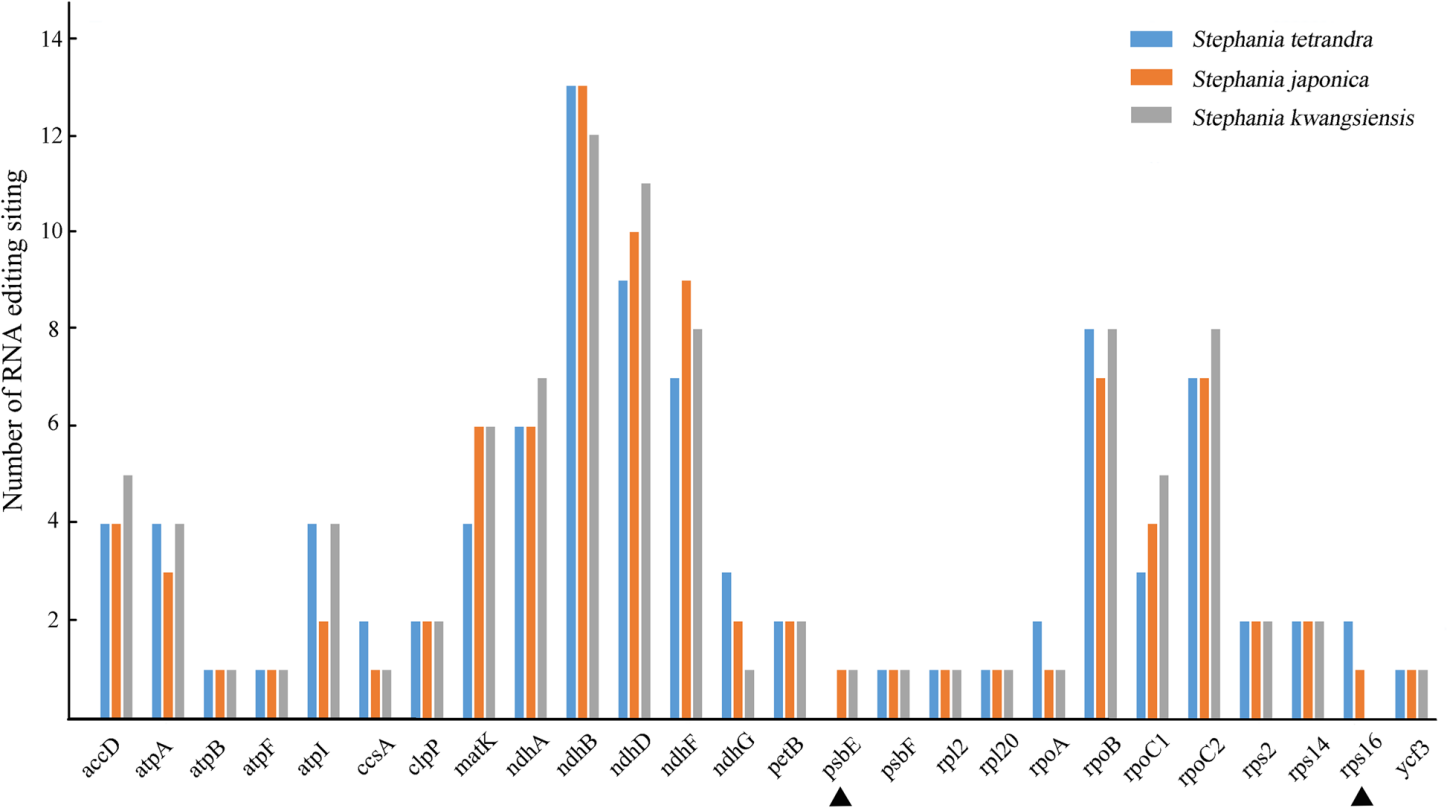
Number of the RNA editing sites in the cp genome of *Stephania tetrandra, Stephania japonica* and *Stephania kwangsiensis* predicted by PREP-Cp program with a cutoff value 0.8

A total of 92 potential RNA editing sites have been predicted in 25 protein-coding genes of the cp genome of *S. tetrandra* (Fig. 3). Among 92 RNA editing sites, 24 codons were observed to be edited at the first nucleotide position whereas 66 codons were found to be edited at the second nucleotide position, and 2 codons edited both at the first and second nucleotide (Table 4). All of the detected codon changes in the cp genome of *S. tetrandra* showed C to T conversions. The *ndhB* gene had the largest number of RNA editing sites (13 editing sites), followed by *ndhD* (9 editing sites), while *atpF*, *atpB*, *psbF*, *rpl20*, *ycf3* and *rpl2* exhibited only one editing site in *S. tetrandra*. These editing sites resulted in 12 kinds of amino acid conversions (Table 4). The conversions of *H to Y, P to S, L to F, R to W, R to C* were due to codons edited at the first nucleotide position, while the *S to L, P to L, S to F, T to M, A to V, T to I* conversions were because of codons edited at the second nucleotide position. Only the conversion of *P to F* was due to codons edited at the first and second nucleotide positions. The conversion of serine to leucine *(S to L*) was the most abundant kind of conversion, accounting for 42.4%, followed by proline to leucine (*P to L*) and histidine to tyrosine (*H to Y*) accounted for 12.0% respectively. Furthermore, the predicted RNA editing sites in the cp genome of *S. japonica* and *S. kwangsiensis* showed similar results with that of *S. tetrandra*, with the number of 91 and 96 respectively (Fig. 3). It is important to note that the RNA editing site was lost in *psbE* gene of *S. tetrandra* and *rps16* gene of *S. kwangsiensis*, which would bring crucial impacts on the translation and protein activity of these genes (Fig. 3). Since the close correlation between RNA editing sites and nucleotide substitution of protein coding genes, we performed further analysis to investigate the synonymous substitutions (Ks) and non-synonymous substitutions (Ka) of protein coding genes with abundant RNA editing sites (Table 5). The Ka/Ks ratios of most genes (23/25) in *S. tetrandra* were less than 0.5, suggesting an obvious purifying selection pattern. Particularly, both of the *petB* and *psbF* gene even exhibited a Ka/Ks value of 0, showing the two genes were possibly under strong purifying selection pressure (Table 5). However, *atpF* showed a Ka/Ks value greater than 1.00, which indicated it is under diversifying selection pattern and would play significant roles during pressure evolution in *S. tetrandra* (Table 5). The RNA editing site and nucleotide substitution of protein-coding genes provide valuable information for understanding of missense mutations in the cp genome of *S. tetrandra*.

**Table 4:** Amino acid conversion frequency of protein coding gene of three *Stephania* species

Amino acid conversion	Edited position	Number and percentage		
		*S. tetrandra*	*S. japonica*	*S. kwangsiensis*
S-L	Second nucleotide	39/42.4%	34/37.8%	34/36.2%
P-L	Second nucleotide	11/12.0%	13/14.4%	14/14.9%
H-Y	First nucleotide	11/12.0%	10/11.1%	11/11.7%
L-F	First nucleotide	4/4.3%	5/5.6%	3/3.2%
S-F	Second nucleotide	5/5.4%	5/5.6%	5/5.3%
T-M	Second nucleotide	4/4.3%	4/4.4%	4/4.3%
A-V	Second nucleotide	3/3.3%	4/4.4%	5/5.3%
P-S	First nucleotide	2/2.2%	4/4.4%	4/4.3%
R-W	First nucleotide	5/5.4%	4/4.4%	5/5.3%
T-I	Second nucleotide	4/4.3%	5/5.6%	5/5.3%
R-C	First nucleotide	2/2.2%	0	2/2.1%
P-F	First and second nucleotide	2/2.2%	2/2.2%	2/2.1%

**Table 5:** The value of Ka/Ks in 25 protein coding genes with RNA editing sites in *S. tetrandra* voucher ZJ

Gene	Number of RNA editing sites	Non-synonymous substitutions (Ka)	Synonymous substitutions (Ks)	Ka/Ks
***ndhB***	13	0.0035	0.0107	0.3271
***ndhD***	9	0.0106	0.0947	0.1119
***rpoB***	8	0.0045	0.0613	0.2887
***rpoC2***	7	0.0177	0.0615	0.0732
***ndhF***	7	0.0202	0.0181	0.1869
***ndhA***	6	0.0074	0.0927	0.0798
***matK***	4	0.0373	0.0948	0.3936
***atpA***	4	0.0061	0.0617	0.0989
***accD***	4	00159	0.0444	0.3581
***atpI***	4	0.0054	0.0385	0.1403
***rpoC1***	3	0.0071	0.0595	0.1193
***ndhG***	3	0.0101	0.0734	0.1376
***ccsA***	3	0.0239	0.1146	0.2086
***rpoA***	2	0.0207	0.0841	0.2461
***rps2***	2	0.0037	0.0824	0.0449
***clpP***	2	0.0022	0.0554	0.0397
***rps14***	2	0.0131	0.0453	0.2892
***rps16***	2	0.0047	0.0295	0.1593
***petB***	2	0.08	0	0
***atpB***	1	0.0036	0.0643	0.0559
***atpF***	1	0.2122	0.1658	1.2799
***rpl2***	1	0.0091	0.0108	0.8426
***rpl20***	1	0.0116	0.0486	0.2387
***ycf3***	1	0.0078	0.0423	0.1844
***psbF***	1	0	0	0

### SSRs and long repeats analysis

**Fig. 4:**
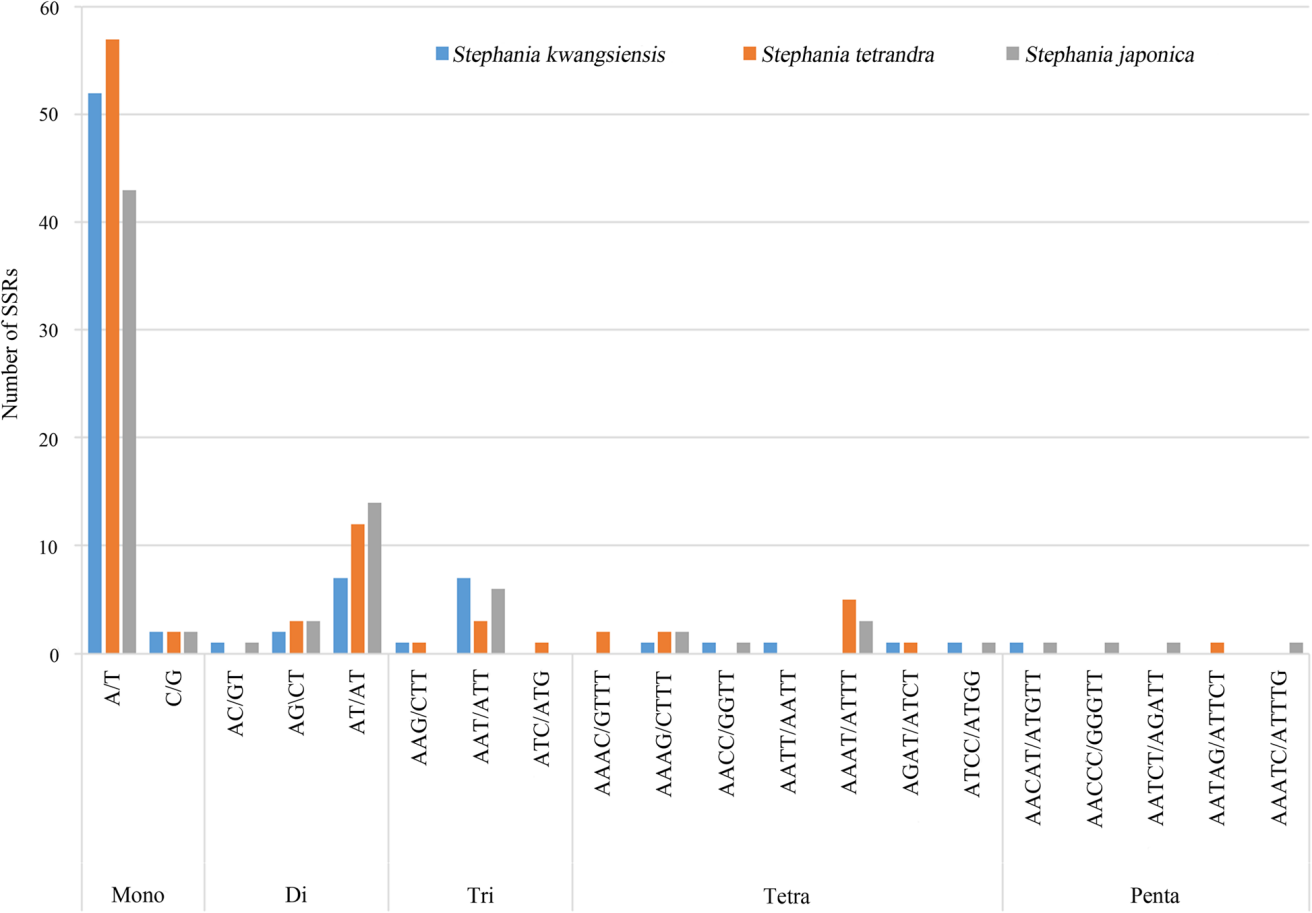
Number of different types of SSRs in the cp genomes of *Stephania tetrandra, Stephania japonica* and *Stephania kwangsiensis,* setting parameters as 10 for mononucleotide SSRs (Mono), 5 for dinucleotide SSRs (Di), 4 for trinucleotide SSRs (Tri), 3 each for tetranucleotide (Tetra), pentanucleotide (Penta) and hexanucleotide (Hexa) SSRs

A total of 90, 80, 78 simple sequence repeats (SSRs) have been detected in the cp genomes of *S. tetrandra*, *S. japonica* and *S. kwangsiensis*, respectively (Fig. 4). In the cp genomes of the three *Stephania* plants, the mononucleotide repeats were consisted of 10–18 repeat units, dinucleotide repeats were consisted of 5–10 repeat units, trinucleotide repeats were consisted of 4–5 repeat units, tetranucleotide repeats were consisted of 3–4 repeat units, and pentanucleotide repeats contained 3 repeat units (Tables S1, S2, S3). SSRs composed of A/T were more abundant than those containing G or C in the cp genomes of the three *Stephania* plants. The mononucleotide repeat A/T was the most abundant, encountering 57 times in *S. tetrandra*, 52 times in *S. kwangsiensis* and 43 times in *S. japonica,* respectively (Table 6). And the second most repeat was the dinucleotide SSR AT/AT. The number and type of pentanucleotide SSRs in the cp genomes of the three plants exhibited some differences. The *S. japonica* had four different types of pentanucleotide SSRs (AACAT/ATGTT, AACCC/GGGTT, AATCT/AGATT, AAATC/ATTTG) whereas the *S. tetrandra* and *S. kwangsiensis* had only one type (AATAG/ATTCT in *S. tetrandra*, AACAT/ATGTT in *S. kwangsiensis*). The number of all types of pentanucleotide SSR was only one. No hexanucleotide SSR has been detected in the cp genomes of the three species. Compared with the IR region, SSRs were mainly distributed in LSC and SSC regions. These SSR markers would be useful for genetic diversity investigation and development of conservation strategy for *Stephania* plants.

**Table 6:** The SSR types of the three *Stephania* plants

SSR type	Repeat unit	Amount		
		*Stephania kwangsiensis*	*Stephania tetrandra*	*Stephania japonica*
Mono	A/T	52	57	43
	C/G	2	2	2
Di	AC/GT	1	0	1
	AG/CT	2	3	3
	AT/AT	7	12	14
Tri	AAG/CTT	1	1	0
	AAT/ATT	7	3	6
	ATC/ATG	0	1	0
Tera	AAAC/GTTT	0	2	0
	AAAG/CTTT	1	2	2
	AACC/GGTT	1	0	1
	AATT/AATT	1	0	0
	AAAT/ATTT	0	5	3
	AGAT/ATCT	1	1	0
	ATCC/ATGG	1	0	1
Penta	AACAT/ATGTT	1	0	1
	AACCC/GGGTT	0	0	1
	AATCT/AGATT	0	0	1
	AATAG/ATTCT	0	1	0
	AAATC/ATTTG	0	0	1

**Fig. 5:**
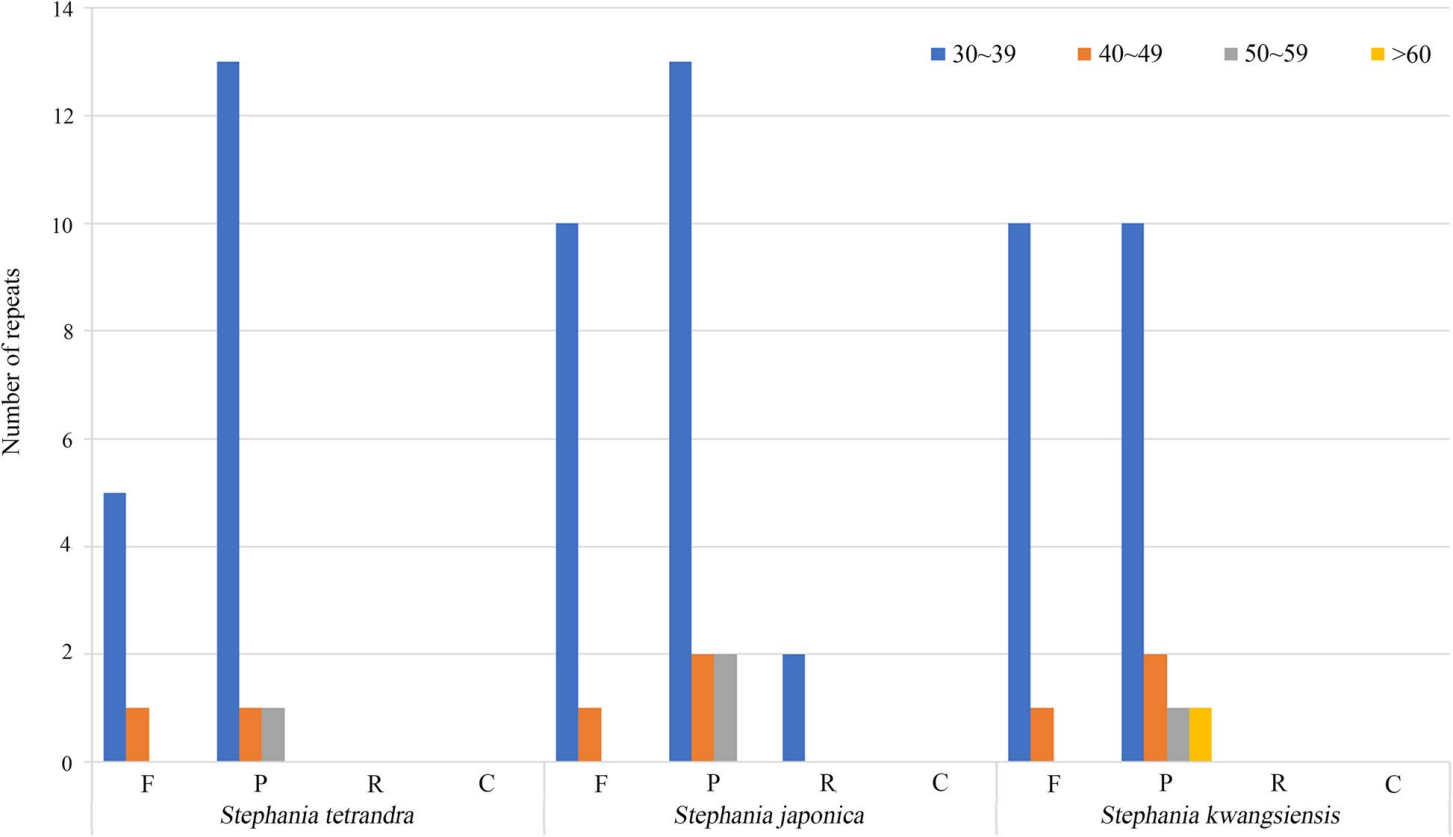
The analysis of the number and length of the long repeats identified from *Stephania tetrandra, Stephania japonica* and *Stephania kwangsiensis* chloroplast genomes. The type of long repeats contains forward (F), palindromic (P), reverse (R) and complement (C). The hamming distance of 3, the minimal repeats of 30 and the maximum repeats of 50 were applied during the calculating process

REPuter program was used to analyze long repeat sequences in the cp genomes of the three *Stephania* plants. The results indicated three types of repetition (forward, reverse and palindrome), with a length of exceeding 30 bp. After removing the two IR regions, which were the largest palindromic repeats in the chloroplast genome, we identified a total of 21 long repeat sequences in *S. tetrandra* cp genome, including 6 forward and 15 palindromic repeats (Fig. 5). *S. japonica* have been identified 11 forward, 17 palindromic and 2 reverse repeats in cp genome, while *S. kwangsiensis* contained 11 forward, 14 palindromic repeats in cp genome. Most of the long repeats are 30–39 bp in length. The palindromic repeat was the most abundant long repeat type and no complimentary repeat has been found in the cp genomes of the three *Stephania* plants. Furthermore, these detected repeats were mainly distributed in LSC region of *Stephania* species.

### IR expansion and contraction

**Fig. 6:**
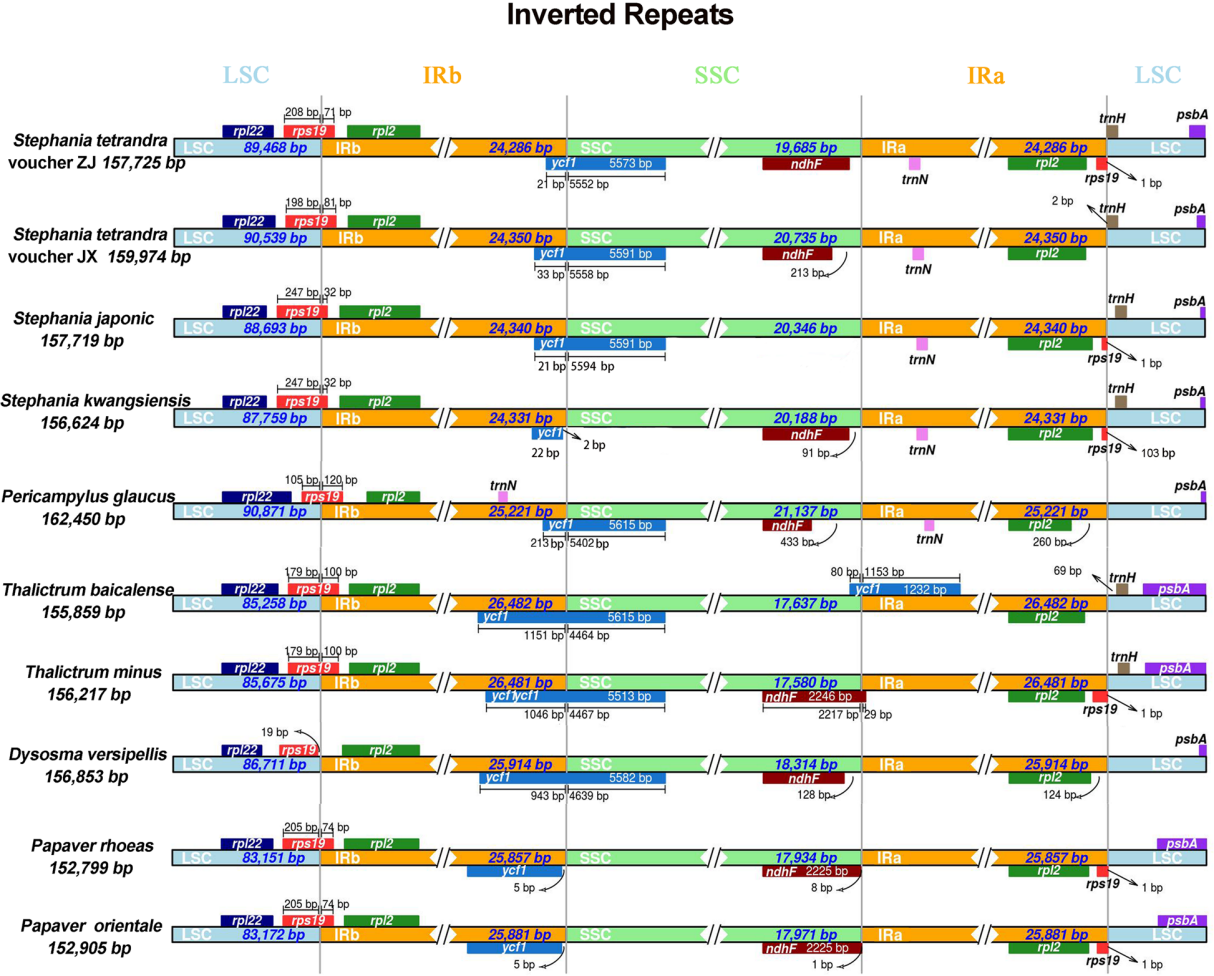
Comparison of junctions between the large single-copy (LSC), small single-copy (SSC) and inverted repeat (IR) regions among 10 chloroplast genomes, including four *Stephania* plants, one closely related species in family of Menispermaceae, and five species from Berberidaceae, Ranunculaceae and Papaveraceae. The numbers of above the gene features indicate the distance between the ends of genes and border sites

The expansion and contraction of IR region is the most common evolutionary event in genome evolution, which is considered to be one of the reasons for the length change between different genomes [26]. Therefore, the junctions of LSC/IR and IR/SSC are sometimes regarded as an index of chloroplast genome evolution. To evaluate the potential impact of the junction changes in the chloroplast genomes of Stephania species, we compared the LSC/IRb/SSC/IRa boundary regions of four Stephania plants and other closely related species from Menispermaceae, Berberidaceae, Ranunculaceae and Papaveraceae to determine the unique and common cp genome features (Fig. 6). The length of the IR regions was similar within the *Stephania* plants, ranging from 24,286 bp in *S. tetrandra* voucher ZJ to 24,350 bp in *S. tetrandra* voucher JX (Fig. 6). However, the *Stephania* species harbored the shortest IR regions compared with other plants from Ranunculales with the longest IR region of 26,482 bp in *Thalictrum baicalense*. Our results indicated that the locating position of *ycf1* gene was highly variable at the boundary of IRB-SSC region in *Stephania* species. The length of *ycf1* gene in *S. tetrandra* voucher ZJ was 18 bp shorter than that of *S. tetrandra* specimen from Jiangxi Province, with embedded length in IRB region of 21 and 33 bp, respectively (Fig. 6). The variation in gene length and expansion extent of *ycf1* from two *S. tetrandra* vouchers indicated potential markers for further geographical origin discrimination. However, the contraction of IR region in *S. kwangsiensis* led to the formation of pseudogene *ycf1*, which was located completely in the IRB region with 2 bp away from the SSC-IRB border. It is worth noting that the overlap between *ycf1* gene and the IRB region varied from 213 to 1151 bp in the cp genome from Menispermaceae, Berberidaceae and Ranunculaceae plants, which was significantly longer than that of *Stephania* species (Fig. 6). The junction between LSC and IRB region was located in the intergenic *rps19* in most species of Ranunculales except *Dysosma delavayi*, in which the *rps19* gene was completely encoded in LSC region and exhibited a 19 bp distance to the junction of the LSC/IRB region (Fig. 6). The length of IRB expansion to *rps19* varied among the Ranunculales species ranging from 32 bp to 120 bp. However, a highly conserved length of *rps19* gene (279 bp) was predicted in most of these Ranunculales plants except *Pericampylus glaucus* encoded a *rps19* gene of 225 bp (Fig. 6). Beside *ycf1* and *rps19*, other genes of *ndhF*, *rpl2*, *rpl22* and *trnH(GUG)* were also found in the the LSC/IR and SSC/IR borders among the cp genomes from Ranunculales species.

### Substitutions and InDels analysis

SNP (single nucleotide polymorphism) is a genetic marker formed by single nucleotide variation in the genome, which generally refers to single nucleotide variation with mutation frequency greater than 1% [27]. Using *Pericampylus glaucus* as reference, SNPs and InDels variations in cp genomes of the four *Stephania* plants have been analyzed. The analysis results revealed that transition substitution was more than transversion substitution in the cp genome of the four *Stephania* plants, which caused the values of transition to transversion ratio (Ts/Tv) much higher than 1 (Table 7). Moreover, the SNP sites mainly distributed in LSC region, followed by SSC region and IR region.

**Table 7:** Comparison of substitutions and InDels in four *Stephania* species

**Comparative analyses of substitutions**									
**Species**	**Region**	**Transition Substitutions**	**Transversion Substitutions**					**Total Number of SNP sites**	**Ts/Tv**
		A/G	C/T	A/T	A/C	C/G	G/T		
***S. tetrandra*** **voucher ZJ**	Large single copy	158	173	13	61	15	35	455	2.6
***S. tetrandra*** **voucher JX**		177	207	20	64	19	40	527	2.7
***S. japonica***		242	245	28	69	26	60	670	2.5
***S. kwangsiensis***		228	239	39	87	25	61	679	2.2
***S. tetrandra*** **voucher ZJ**	Inverted repeat	8	9	0	3	2	1	23	2.1
***S. tetrandra*** **voucher JX**		10	7	1	5	1	3	27	1.7
***S. japonica***		10	11	1	4	2	5	33	1.9
***S. kwangsiensis***		9	13	1	5	1	5	34	1.8
***S. tetrandra*** **voucher ZJ**	Small single copy	43	38	5	19	4	18	127	2.0
***S. tetrandra*** **voucher JX**		64	61	15	21	9	10	180	2.2
***S. japonica***		68	92	21	25	11	21	238	2.2
***S. kwangsiensis***		73	66	13	27	7	24	210	1.9
**Comparative analyses of InDels sites**									
**Species**	**Large single copy**		**Inverted repeat**				**Small single copy**		
	No’s of Indels	InDel average length	No’s of Indels		InDel average length		No’s of Indels	InDel average length	
***S. tetrandra*** **voucher ZJ**	11	88.636	5		104.400		5	28.200	
***S. tetrandra*** **voucher JX**	13	42.581	2		87.300		5	28.200	
***S. japonica***	19	37.421	6		89.500		5	28.200	
***S. kwangsiensis***	21	34.571	4		134.250		5	23.400	

A total of 21 InDels in the protein-coding sequences of the cp genome from *S. tetrandra* voucher ZJ were identified, including 11 InDels locating in LSC region, 5 InDels locating in SSC region, and 5 InDels locating in one IR region. However, only 20 InDel substitutions were revealed in the cp genome coding regions of *S. tetrandra* voucher JX, with the number of 13, 2 and 5 distributing in the LSC, SSC and IR region, respectively (Table 7). In addition, a total of 101 InDels have been identified in four *Stephania* plants, including 64 in the LSC region, 17 in the SSC region and 20 the in the IR region, respectively. The highest number of InDels substitutions was observed in *S. japonica* and *S. kwangsiensis* (30), while the lowest number was detected in *S. tetrandra* voucher JX (20). The LSC region of cp genome harbored the largest number of InDel sites in *Stephania* species. Furthermore, *S. tetrandra* voucher ZJ showed the largest InDel average length (88.636 bp) in LSC region, while *S. kwangsiensis* exhibited the largest InDel average length in IR region (134.250 bp). The Indel average length in SSC region ranged from 23.4 bp to 28.2 bp in the cp genome of four *Stephania* species, which represented the shortest one (Table 7).

### Identification and analysis of divergence regions

**Fig. 7:**
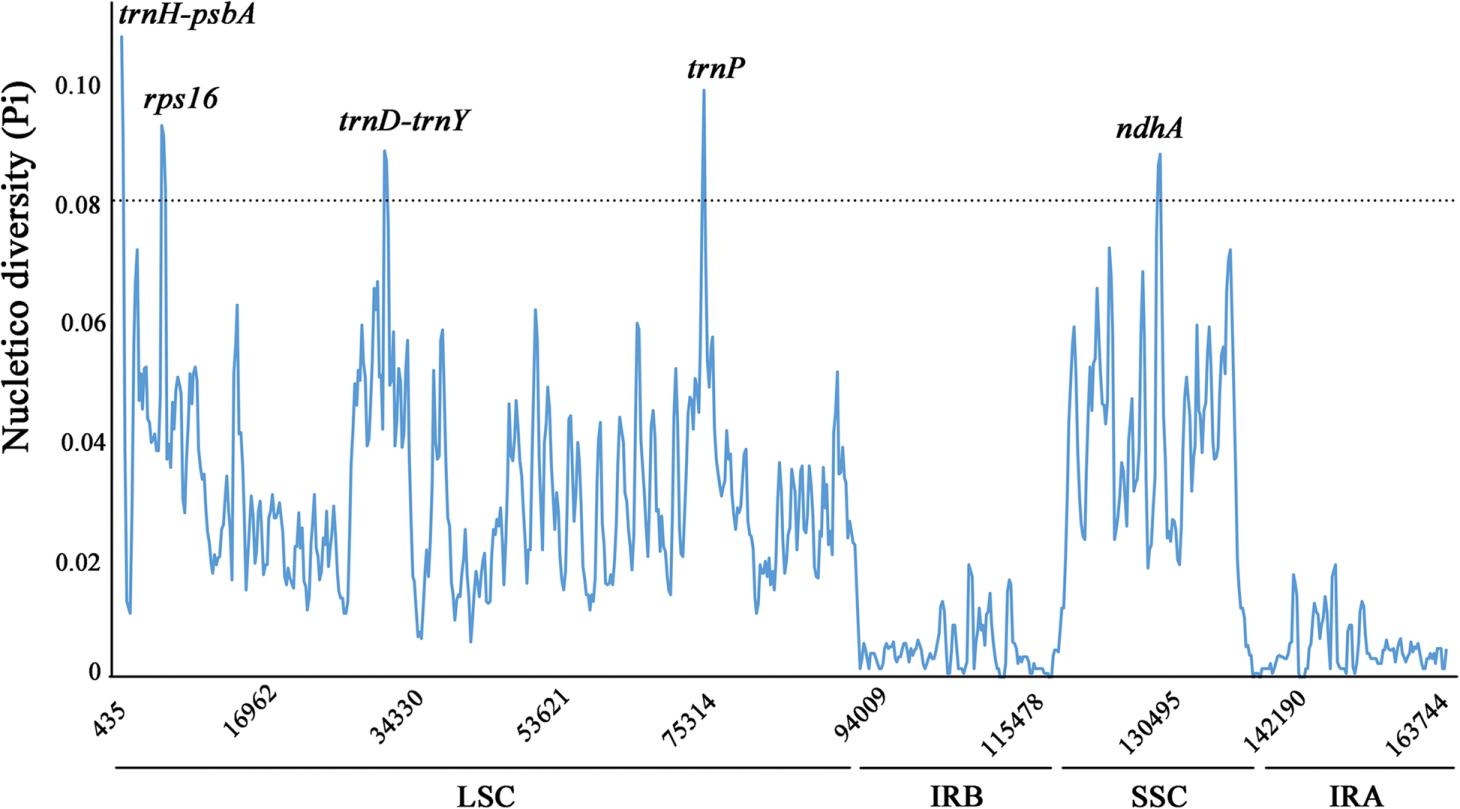
Nucleotide diversity (Pi) analysis for chloroplast genomes from the *Staphania* plants. Sliding window length was 800 bp and step size was selected as 200 bp

In order to evaluate the sequences divergence level, the complete cp genomes of the four *Stephania* plants have been multiple aligned and used DnaSP software to calculate nucleotide variability (Pi). A total of 5 mutational hotspot loci with high Pi value (> 0.08) have been screened out in the *Stephania* species, including 3 intergenic regions (*trnH-psbA, trnD-trnY, trnP)* and two protein coding genes (*rps16* and *ndhA*) (Fig. 7). These high Pi value regions suggested to be divergence regions in the complete cp genome of the four *Stephania* plants. The highest Pi value was identified in the region of *trnH-psbA* (0.10639), followed by that from *trnP* region (0.0975). The most conserved region with the lowest pi value was found in the IR regions, further confirming that the IR regions were highly conserved in the chloroplast genomes of *Stephania* plants.

To further evaluate the diversities of 5 mutational hotspots among *Stephania* plants, we carried out a comparative analysis to determine the numbers of SNP sites and Gaps, as well as the values of non-synonymous to synonymous substitution (Ka/Ks) ratio. Multiple SNP sites were identified in the 5 mutational hotspots, with the highest SNP number of 136 in *rps16* region (Table 8). In addition, a large number of Gaps were detected in the 5 mutational hotspots with the length ranging from 7 to 83 bp, which led to different mutational hotspot length in the four *Stephania* plants. The Ka/Ks ratio analysis demonstrated that the 5 mutational hotspots were suffering different selection pressures in the *Stephania* species. Most of the mutational hotspots exhibited a Ka/Ks ratio value much smaller than 1.00, indicating a purifying selection pattern. However, the Ka/Ks values of *trnH-psbA* in *S. tetrandra* voucher ZJ (1.0892), *trnD-trnY* in *S. japonica* (1.4076)*, S. kwangsiensis* (1.1759), *trnP* in *S. tetrandra* voucher ZJ (1.1528), *S. japonica* (1.1237), *S. kwangsiensis* (1.5410) and *ndhA* in *S. tetrandra* voucher ZJ (1.3464), *S. japonica* (1.0783) were greater than 1.00, demonstrating that these genes were under diversifying selection pattern and more sensitive to the environment pressure. It is interesting to note that the Ka/Ks values of these identified mutational hotspots were all less than 1.00 in *S. tetrandra* voucher JX, suggesting potential population genetic variations between the two *S. tetrandra* species from Jiangxi and Zhejiang Province (Table 8). These results indicated the mutational hotspots could provide potential molecular markers to resolve the difficulties in species identification of *Stephania* species. According to the conserved regions surrounding the variable sties of the mutational hotspots, 5 pairs of PCR primers were designed to amplify the potential molecular markers (*trnH-psbA*, *rps16*, *trnP*, *ndhA* and *trnD-trnY*), which provided an effective tool for species identification and population genetic investigation in *Stephania* plants (Table 9). Furthermore, the significant SNP and Gap differences on five mutational regions observed between *S. tetrandra* voucher ZJ and voucher JX demonstrated high genetic differentiations among *S. tetrandra* populations from different regions, suggesting the potential of developing informative interspecifically variable sites as markers to verify its geographic origin.

**Table 8:** Multiple analysis of the mutational hotspots in four *Stephania* plants

Mutational hotspots	Species	Length	Number of SNP sites	Total length of Gaps	Ka/ks
***trnH-psbA***	*S. tetrandra* voucher ZJ	543 bp	/	/	1.0892
	*S. tetrandra* voucher JX	597 bp	62	30	0.9363
	*S. japonica*	594 bp	83	29	0.7585
	*S. kwangsiensis*	556 bp	121	31	0.8556
***rps16***	*S. tetrandra* voucher ZJ	1146 bp	/	/	0.5510
	*S. tetrandra* voucher JX	1206 bp	97	26	0.5689
	*S. japonica*	1124 bp	83	29	0.7639
	*S. kwangsiensis*	827 bp	136	83	0.7030
***trnD-trnY***	*S. tetrandra* voucher ZJ	404 bp	/	/	0.7224
	*S. tetrandra* voucher JX	475 bp	65	14	0.8217
	*S. japonica*	481 bp	70	8	1.4076
	*S. kwangsiensis*	481 bp	57	9	1.1759
***trnP***	*S. tetrandra* voucher ZJ	662 bp	/	/	1.1528
	*S. tetrandra* voucher JX	687 bp	77	28	0.9143
	*S. japonica*	673 bp	91	18	1.1237
	*S. kwangsiensis*	616 bp	106	32	1.5410
***ndhA***	*S. tetrandra* voucher ZJ	531 bp	/	/	1.3464
	*S. tetrandra* voucher JX	492 bp	97	8	0.7692
	*S. japonica*	507 bp	84	7	1.0783
	*S. kwangsiensis*	492 bp	91	21	0.9638

Gaps consists of the insertion and deletion of single bases and fragments

**Table 9:** PCR primers designed according to the mutational hotspots within four *Stephania* species

Mutational hotspots	PCR primers	Expected length
***trnH-psbA***	F:CGCCGTAGTAAATAGGAGA	860 bp
	R:TCATCAACCGYGCTAACCT	
***rps16***	F:RATACAATAAGCAAGCTC	957 bp
	R:TCCCRAAACAAGAAAACG	
***trnD-trnY***	F:GTGCTCTGACCGATTGAACT	525 bp
	R:GGCAATATGTCTACGCTGGT	
***trnP***	F:TAGGTAGGGATGACAGGA	918 bp
	R:GACCCGAACCATAGAGTA	
***ndhA***	F:CRAATCCMAAATTAGACCA	668 bp
	R:GACGCTTAGGAACACCAA	

### Phylogenetic analysis

**Fig. 8:**
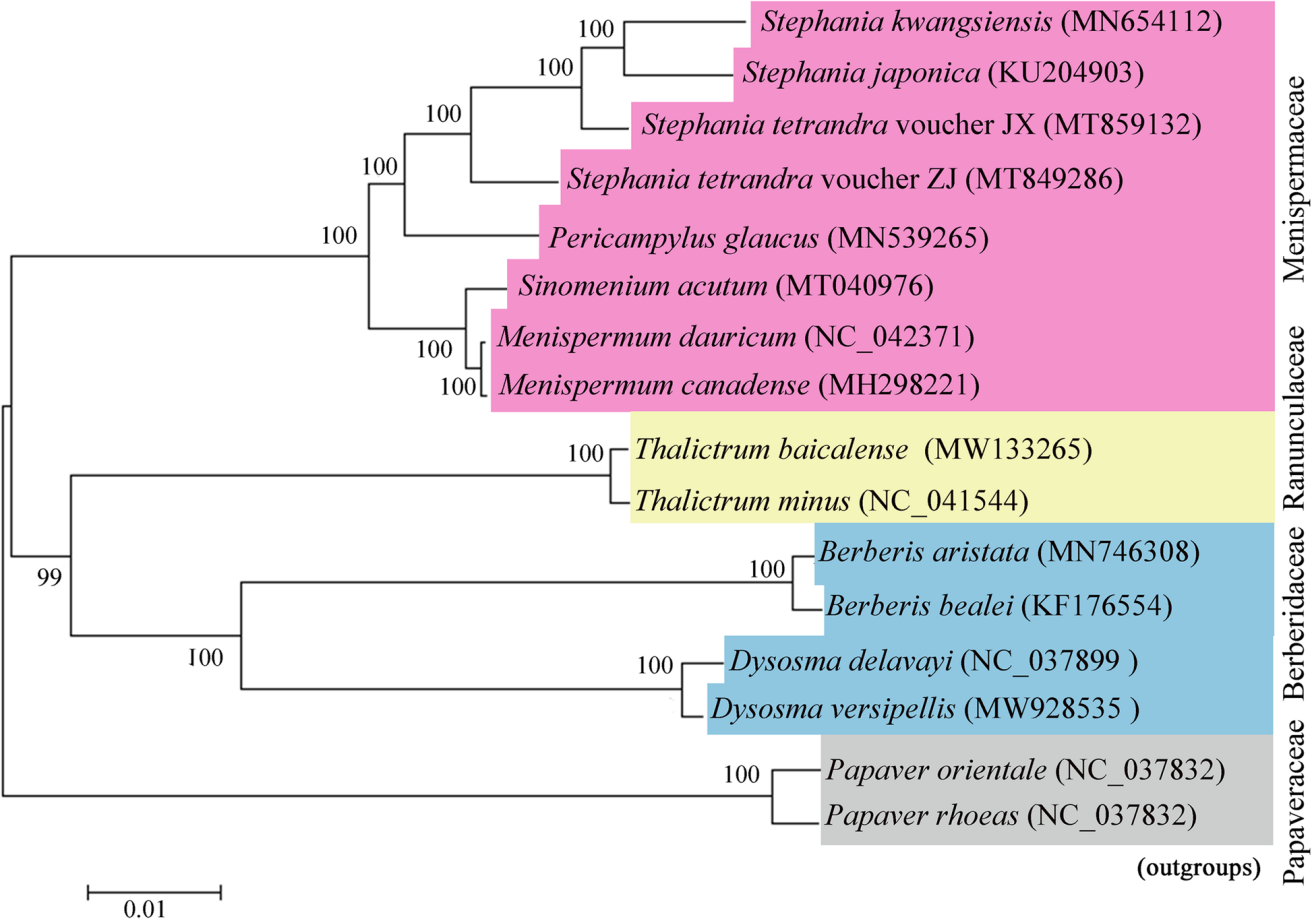
Phylogenetic relationships based on the conserved chloroplast protein coding genes from four *Stephania* plants and other representative Ranunculales species using maximum likelihood (ML) method. The number on each node represents the bootstrap value from 500 replicates. *Papaver orientale* and *Papaver rhoeas* were set as the outgroups

The conserved chloroplast genomes have been indicated as effective approach for the phylogenetic relationship studies of plants from different taxa [28]. The phylogenetic tree was constructed using maximum likelihood (ML) method for Ranunculales plants based on coding sequences of 76 protein-coding genes, including 8 species from family Menispermaceae, 2 species from family Ranunculaceae and 4 species from family Berberidaceae. *Papaver orientale* and *P. rhoeas* were chosen as the outgroups for phylogenetic analysis. As shown in Fig. 8, all nodes received high support values in the ML tree, and it was congruent with those obtained from the previous study. The phylogeny analysis showed *S. japonica* and *S. kwangsiensis* consisted into a stable monophyletic group with high bootstrap values, which exhibited a stable sister relationship with *S. tetrandra* voucher JX and voucher ZJ, indicating a close genetic relationship among the four *Stephania* plants. Interestingly, *S. tetrandra* voucher ZJ and voucher JX failed to clustered into the same branch, revealing obvious discrepancies on cp genomes of *S. tetrandra* from different regions (Fig. 8). In addition, the *Stephania* species formed a clade with *Pericampylus glaucus* with strong statistical support, indicated a relatively close relationship between the genus *Stephania* and *Pericampylus* in *Trib. Menispermeae*. Moreover, the species from the genus of *Menispermum* and *Sinomenium* combined together to form a robust monophyletic clade, which showed sister relationship to the group of *Stephania* and *Pericampylus* plants. The entire subclade comprising of species from family of Berberidaceae and Ranunculaceae grouped with the clade of family Menispermaceae, which were clustered together to form the monophyletic group of core Ranunculales (Fig. 8). The complete chloroplast sequence of *S. tetrandra* voucher ZJ could provide useful information for clarifying the phylogenetic relationship of family Menispermaceae as well as the classification of subgenera and species groups in *Stephania*.

## Discussion

In this study, we sequenced and assembled the complete cp genome of *S. tetrandra* from Zhejiang province and analyzed its codon usage bias and RNA editing sites. We also conducted a comparative analysis on the RNA editing sites, codon usage bias, repeat sequences, substitutions and InDels events within the complete cp genomes of the *Stephania* plants and constructed the phylogenetic tree. Our results provide basic data for further studies on the identification and phylogenetic relationship of Menispermaceae plants. The genome database of *S. tetrandra* voucher ZJ would be also beneficial for the subsequent specific molecular species identification and population genetic analysis from different regions in *Stephania* plants.

The *Stephania* is a genus of Menispermaceae, with three subgenera of *Subgen. Botryodiscia*, *Subgen. Stephania* and *Subgen. Tuberiphania* [29]. *S. tetrandra* is the only plant that belonging to the *Subgen. Botryodiscia* and has attracted considerable interest owing to its significant medicinal values. The species of *S. japonica* and *S. kwangsiensis* were classified to the *Subgen. Stephania* and *Subgen. Tuberiphania*, respectively. The ML trees constructed based on *ITS* and *trnL-F* in previous reports showed conflicting results in the determination of the relationship among the three subgenera of the genus *Stephania*. The ML trees based on *ITS* showed that *Subgen. Tuberiphania* species formed a branch and *S. tetrandra* clustered with the *Subgen. Tuberiphania* species to form a monophyletic group [12]. However, the ML trees of *trnL-F* indicated that the clade of *Subgen. Stephania* species exhibited a sister relationship with that of *Subgen. Tuberiphania* species, and *S. tetrandra* clustered with *Perichasma laetificata* and the combined group of *Subgen. Stephania* and *Subgen. Tuberiphania* species [12]. Besides molecular investigation strategy, the chemical composition analysis could also provide valuable information for the relationship research within *Stephania* species. For instance, *S. cepharantha* belonging to the *Subgen. Tuberiphania* showed no chemical compounds of L-tetrahydropalmatine and tetrandrine, which exhibited similar chemical compositions with plants from *Subgen. Stephania* [30]. The chemical analysis indicated *S. cepharantha* may be a transitional type between *Subgen. Tuberiphania* and *Subgen. Stephania* [30]. In addition, hasubanan alkaloids were the unique alkaloids in plants of genus *Stephania*, which were considered to be of great significance in the study of classification and evolution of *Stephania* [31]. Most of plants classified to *Subgen. Stephania* and *Subgen. Tuberiphania*, which are rich sources of hasubanan alkaloids. However, there are no such chemical in *S. tetrandra*, indicating a closer relationship between *Subgen. Stephania* and *Subgen. Tuberiphania*. Our result showed a close phylogenetic relationship between *Subgen. Stephania* and *Subgen. Tuberiphania*, which was consistent with the previous ML analysis based on *trnL-F* marker. Furthermore, both of the *S. tetrandra* samples from Jiangxi and Zhejiang Province respectively clustered with combined clade of *Subgen. Stephania* and *Subgen. Tuberiphania*, further confirming the reasonability of dividing the *S. tetrandra* as a separate subgenus (Fig. 8). The chloroplast genome of *S. tetrandra* voucher ZJ provides a valuable reference for the study of phylogenetic relationship within *Stephania* genus, which required more accurate and abundant chloroplast genome information from *Stephania* plants.

Codon usage bias and RNA editing were important events for the protein-coding genes in plant cp genomes, which was closely related with the molecular evolutionary phenomena such as mutation, selection, and random genetic drift. Qin et al. (2013) suggested that the synonymous codon usage bias was correlated with intron number in plants, which may be due to DNA methylation [32]. The GC content in codon positions was also indicated as an important role during the evolution of genomic structure and one of the major factors in the codon usage biases shaping [33]. Our results demonstrated that among the 31 codons with RSCU value higher than 1, 29 codons ended with A or U. However, most of the codons (28) with RSCU value less than 1 ended with G or C. This result indicated that *S. tetrandra* preferred to use synonymous codons with a third base of A or U, which was similar with that of *Fagopyrum dibotrys* and *Salix wilsonii* [34, 35]. The RNA editing process is an essential maturation mechanism to avoid incorrect RNA mutations and is widespread in the chloroplast genome of plants [36]. The RNA editing sites analysis of the cp genome is vital to understand the correct translation process and mutations of genes [14]. In our study, 92 RNA editing sites were predicted in 25 protein-coding genes of the cp genome of *S. tetrandra*, which may affect the structure and function of proteins. Previous studies have shown that the deletion editing sites of psbF-26 in *Arabidopsis* would affect the assembly of PSII complex seriously [37]. In addition, the RNA editing of *rpoA* and *clpP* chloroplast transcripts by CLB19 played crucial roles for the s essential for correct chloroplast development and phenotype development [38]. Our results found the RNA editing sites of *psbE* and *rps16* genes were lost in certain *Stephania* species (Fig. 3). However, the functional changes caused by the deletion of RNA editing sites in *Stephania* plants remained unknown and required further exploration.

Mutational hotspots from chloroplast genome have been demonstrated as potential molecular markers that might be developed for phylogenetic relationships analysis and identification among closely related species. Jiao et al. (2019) have found seven variable regions from the chloroplast genome of *Pterocarpus* species, and suggested *ycf1b* could be applied as a high-resolution DNA barcode for species identification of *Pterocarpus* wood [21]. In addition, Bi et al. (2018) selected ten regions with relatively high variability from the cp genome of *Fritillaria* species and demonstrated that the regions of *ycf1a* and *ycf1b* showed highest variability, providing valuable resources for the study of species identification, phyletic evolution, breeding direction and population genetics [39]. In this study, we also calculated nucleotide variability (Pi) using sliding window analysis and identified 5 high Pi value regions and designed five pairs of primers. These markers were believed to be potential divergence regions in the complete cp genome of the four *Stephania* plants, providing the basis for subsequent molecular identification. The *trnH-psbA* region had the highest Pi value and the *trnP* also exhibited high variability in the cp genomes which might be developed as a molecular marker for subsequent research on resolving the difficulties of identification and phylogenetic relationships analysis of *Stephania* plants. Furthermore, the divergence regions mainly distributed in LSC and SSC region, which was consist with the previous reports of *Chaenomeles* and *Lancea* species [40, 41]. Multiple SNP sites and Gaps were identified in the divergence regions, which provided basic information for the cost-effective, authentic and robust molecular markers design in *Stephania* plants. The Ka/Ks ratio analysis indicated four mutational hotspots (*trnH-psbA*, *trnP*, *trnD-trnY* and *ndhA*) in *S. tetrandra* voucher ZJ were suffering diversifying, which would be more sensitive to the environment changes (Table 8). It is interesting to note that *S. tetrandra* from different regions exhibited significant differences in the complete genome sequences. For instance, the cp genome size of *S. tetrandra* voucher JX is 2249 bp longer than that of *S. tetrandra* voucher ZJ in our study. Furthermore, the cp genome of *S. tetrandra* voucher JX exhibited insertion of a base A in *ycf15* gene, which caused the base dislocation to move backward and led to the untranslatability of this gene [22]. It is well known that *S. tetrandra* is distributed widely in tropical and subtropical regions of Asia and Africa, generating potentials of great numbers of genetic diversities and unique population structures. Moreover, the geographical origin of herbal medicines is an important factor influencing the quality of the medicinal materials [42]. Our results have revealed the occurrence of genetic variation events of *S. tetrandra* from different regions, which might bring significant differences to the medicinal treatment efficacy and cause health benefits to consumers. Therefore, it is extremely important to develop effective strategy to accurately discriminate the geographical origin of *S. tetrandra*. These results indicated the mutational hotspots of cp genome would be valuable tools for species geographical diversity investigation of *S. tetrandra*, providing a potential approach to determine different original species and ensure its pharmaceutical activity in the market. However, the available complete chloroplast genomes of *S. tetrandra* from different regions are still dramatically insufficient at present, the development of new DNA barcodes based on the chloroplast genome needs further study.

Distribution of repeats in the genome could infer highly polymorphic regions in the genome [17]. SSRs could be developed as molecular markers for species identification, population genetic and phylogenetic relationship analysis [43], In addition, SSR have also been extensively used in genetic analysis, functional gene mapping and quantitative trait locus (QTL) mapping [44]. Chu et al. (2020) have selected 36 SSR primers pairs for identification of cauliflower and broccoli varieties and confirmed that SSR molecular markers could be used to identify cauliflower and broccoli varieties [45]. Huang et al. (2021) developed EST-SSR markers of *Tetraena mongolica,* suggesting that the SSRs might be useful for further study of genetic structure and adaptive evolutionary mechanism and utilization of the germplasm [46]. Moreover, the mononucleotide and dinucleotide repeats were the most and second most abundant SSRs in the cp genomes of two *Caldesia* species [47]. Here, we also found that the mononucleotide repeat A/T was the most abundant SSR and the second most was the dinucleotide SSR AT/AT, which was consist with the previous reports. Furthermore, SSRs have been demonstrated to be generally composed of A or T repeats and infrequently contain C and G repeats in the cp genomes of seven *Populus* species [48]. This result was consisted with our research and the abundance of AT base content in SSRs might be one of the reasons for the high AT content in the chloroplast genome. The chloroplast genomes of the *Stephania* species are rich in SSRs and were good resources to develop SSRs markers for its further studies.

## Conclusion

In summary, in this study we have determined the complete cp genome sequence of *S. tetrandra* voucher ZJ from Zhejiang Province and analyzed its features*.* Comparative analysis between *S. tetrandra* voucher ZJ and other *Stephania* species revealed that the available complete cp genomes from this genus were highly conserved in terms of overall structure, genome size, GC contents, and gene numbers, orders and functions. Comparative analysis involving cp genomes of *Stephania* plants revealed 5 highly variable regions (*trnH-psbA, rps16, trnD-trnY, trnP* and *ndhA*), with multiple SNP sites and Gaps, as well as different non-synonymous to synonymous substitution (Ka/Ks) ratio values. Moreover, five pairs of specific primers targeting the mutational hotspots were accordingly designed, which could be utilized as potential molecular markers for resolving the difficulties in studies regarding species identification, population genetic and phylogenetic analysis in *Stephania* species. The number and types of SSRs, long repeat sequences and the value of Ts/Tv were also detected for developing potential and effective molecular markers. The Maximum likelihood (ML) phylogenetic analysis showed that *S. japonica* and *S. kwangsiensis* consisted into a monophyletic group and exhibited a stable sister relationship with both of the *S. tetrandra* plants from different regions*,* indicating a close genetic relationship among the four *Stephania* species. The cp genome of *S. tetrandra* voucher ZJ provides valuable information for the development of accurate molecular approaches for its geographical origin determination. Our comprehensive analysis of these complete chloroplast genomes will contribute to medicinal resource conservation, genetic diversity, genome evolution and adaptation history, and phylogenetic relationship investigation of *Stephania* plants.

## Methods

### Plant material and DNA extraction

The *S. tetrandra* plant was collected from the mountain in Fuyang District of Zhejiang Province (30°05′38.4″N 119°53′24″E) are identified by Dr. Guanghui Liao of Zhejiang Chinese Medical University. The specimen was deposited in Medicinal Herbarium Center of Zhejiang Chinese Medical University (Herbarium Code: MHCZCMU; Collector and Identifier: Guanghui Liao: voucher number: FJZJ-190826). Modified cetyltrimethylammonium brofmide (CTAB) method was used to extracted total genomic DNA from dried leaves ground with liquid nitrogen [49]. Final DNA quality was assessed by a Nanodrop spectrophotometer (Thermo Fisher Scientific, USA), and DNA integrity was examined by electrophoresis on a 1.0% agarose gel.

### Genome sequencing, assembly and annotation

Total genomic DNA was sequenced using the Illumina Hiseq Platform. The quality of paired-end Illumina reads was assessed with FastQC, and the low-quality reads were removed using Fastp. Then the filtered reads were assembled de novo using metaSPAdes with the complete cp genome of *S. japonica* (NC_029432) as reference and the protein-coding genes, mRNA genes, tRNA genes were annotated by GeSeq annotation tool [50]. The CPGAVAS2 software also used to annotate protein-coding genes [51]. Then the annotation of chloroplast genome was further manually corrected by BLAST. OrganellarGenomeDRAW (OGDRAW) tool was used to draw the circular chloroplast genome map of *S. tetrandra* [52]*.* The fully annotated cp genome was finally deposited at the GenBank database (Accession Number: MT849286).

### Comparative analysis of cp genomes

MEGA 7.0 [53] was used to analyze the genome feature and Codon W software was used to investigate the distribution of codon usage using the RSCU value [54]. To predict the number of RNA editing site, the PREP-Cp program was employed with a cutoff value 0.8 [55]. The IR scope was further applied to analyze the LSC/IRb/SSC/IRa boundary locations in ten Ranunculales species cp genomes [56]. As for the repeats analysis, simple sequence repeats markers in the three *Stephania* species were detected by MISA, setting parameters as 10 for mononucleotide SSRs, 5 for dinucleotide SSRs, 4 for trinucleotide SSRs, 3 each for tetranucleotide, pentanucleotide and hexanucleotide SSRs [57]. Long repeats of four different type (forward (F), palindromic (P), reverse (R), and complementary (C)) were calculated by REPuter, with hamming distance 3, minimal repeats 30 and maximum computed repeats 50 [58].

### Analysis of substitutions and InDels, as well as adaptive evaluation

Using *pericampylus glaucus* (MN539265) as reference sequence, the protein-coding sequences of LSC, SSC and IR regions of the four *Stephania* plants (including *S. tetrandra* voucher JX) were pairwise aligned with corresponding parts of the reference sequence using MAFFT v7.037b [59]. MEGA7.0 was used to determined substitutions numbers and types of the sequences [53], and numbers of InDels events and their average lengths were detected by Dnasp v6 [60]. In order to analyzed the Ka and Ks substitution rates and Ka/Ks ratio, *Pericampylus glaucus* was compared with *S. tetrandra* in 25 protein coding genes. Besides, the Ka/Ks value of 5 mutation hotspots in four *Stephania* plants were also evaluate separately. The alignment was carried out by MAFFT v7.037b [58], and the calculation of the value of Ka/Ks was implemented by DnaSP v6 [60]. The number of mutation sites of the other three *Stephania* species with *S. tetrandra voucher* ZJ as the reference in five mutation regions was analyzed respectively by MEGA 7.0 [53].

### Identification of divergence regions and PCR primers designing

The complete cp genomes of the four *Stephania* plants were multiple aligned by MAFFT v7.037b to evaluate sequence divergence [59], and nucleotide variability (Pi) was calculated by sliding window analysis of DnaSP v6 setting parameters as window length for 800 sites and step size for 200 sites [14]. Specific primers were designed in conserved nucleotide sequences at both ends of mutation hotspots by primer premier 5, and met the conditions of GC content ranging from 40 to 60%, as well as primer length ranging from 15 bp to 30 bp.

### Phylogenetic analysis

*Stephania kwangsiensis* (MN654112), *Stephania japonica* (KU204903), *Sinomenium acutum* (MT040976), *Menispermum canadense* (MH298221), *Menispermum dauricum* (NC_042371), *Pericampylus glaucus* (MN539265), *Stephania tetrandra* (MT859132), *Thalictrum baicalense* (MW133265), *Thalictrum minus* (NC_041544), *Berberis aristata* (MN746308), *Berberis bealei* (KF176554), *Dysosma delavayi* (NC_037899), *Dysosma versipellis* (MW928535), *Papaver orientale* (NC_037832) and *Papaver rhoeas* (NC_037832) were downloaded from NCBI. Seventy-six protein-coding gene sequences that existed in *S. tetrandra* voucher ZJ and other 15 representative species were multiple aligned by MAFFT v7.037b [59] and then analyzed using MEGA 7.0 [53] by maximum-likelihood (ML) method to constructed the phylogenetic tree. ML trees were constructed based on the Kimura 2-parameter model with 500 bootstrap replications. *Papaver orientale* and *Papaver rhoeas* were set as the outgroups.

## Supplementary Information

**Additional file 1: Table S1.** SSRs identified in the cp genome of *Stephania tetrandra.* **Table S2.** SSRs identified in the cp genome of *Stephania japonica.* **Table S3.** SSRs identified in the cp genome of *Stephania kwangsiensis.*

## Data Availability

The chloroplast genome of *Stephania tetrandra* assembled in this study have been deposited in the National Center for Biotechnology and Information (NCBI) under the accession number of MT849286. The raw sequence data reported in this study have been deposited in NCBI with the BioProject, SRA, and BioSample numbers of PRJNA735711, SRR14748481 and SAMN19594056, respectively (https://submit.ncbi.nlm.nih.gov/subs/sra/SUB9808741/overview). The other cp genomes used in this study were downloaded from the NCBI.

## Abbreviations

BLAST: Basic local alignment search tool
cp: Chloroplast
DNA: Deoxyribonucleic acid
IR: Inverted repeat
LSC: Large single copy region
SSC: Small single copy region
SSR: Simple sequence repeats
SNP: Single nucleotide polymorphism
InDel: Insertion and deletion
