# Supplementary material for: Complete chloroplast genome of Stephania tetrandra (Menispermaceae) from Zhejiang Province: insights into molecular structures, comparative genome analysis, mutational hotspots and phylogenetic relationships
Source: BMC Genomics. 2021 Dec 6;22:880. doi: 10.1186/s12864-021-08193-x (PMC8647421; doi:10.1186/s12864-021-08193-x)
Supplement: Supplementary file 1 — Additional file 1: Table S1. SSRs identified in the cp genome of Stephania tetrandra. Table S2. SSRs identified in the cp genome of Stephania japonica. Table S3. SSRs identified in the cp genome of Stephania kwangsiensis. [file 12864_2021_8193_MOESM1_ESM.docx]

**TITLE PAGE**

**Supplementary Tables**

**Title:**

Complete chloroplast genome of *Stephania tetrandra* (Menispermaceae) from Zhejiang Province: insights into molecular structures, comparative genome analysis, mutational hotspots and phylogenetic relationships

**Table S1.** SSRs identified in the cp genome of *Stephania tetrandra.*

**Table S2.** SSRs identified in the cp genome of *Stephania japonica.*

**Table S3.** SSRs identified in the cp genome of *Stephania kwangsiensis.*

**Authors:**

Shujie Dong^1, #^, Zhiqi Ying^1, #^, Shuisheng Yu^2^, Qirui Wang^1^, Guanghui Liao^1^, Yuqing Ge^3^, Rubin Cheng^1^

# These authors contributed equally to this work

**Affiliation:**

^1^ School of Pharmaceutical Sciences, Zhejiang Chinese Medical University, Hangzhou, China

^2^ The Administration Bureau of Zhejiang Jiulongshan National Nature Reserve, Suichang, China

^3^ The First Affiliated Hospital of Zhejiang Chinese Medical University, Hangzhou, China

**Table S1**. SSRs identified in the cp genome of *Stephania tetrandra*.

| Repeats | 3 | 4 | 5 | 6 | 7 | 8 | 9 | 10 | 11 | 12 | 13 | 14 | 15 | Total |
| --- | --- | --- | --- | --- | --- | --- | --- | --- | --- | --- | --- | --- | --- | --- |
| A/T | - | - | - | - | - | - | - | 28 | 8 | 11 | 2 | 5 | 3 | 57 |
| C/G | - | - | - | - | - | - | - | 1 | 1 |  |  |  |  | 2 |
| AG/CT | - | - | 3 |  |  |  |  |  |  |  |  |  |  | 3 |
| AT/AT | - | - | 4 | 3 | 1 | 2 | 2 |  |  |  |  |  |  | 12 |
| AAG/CTT | - | 1 |  |  |  |  |  |  |  |  |  |  |  | 1 |
| AAT/ATT | - | 3 |  |  |  |  |  |  |  |  |  |  |  | 3 |
| ATC/ATG | - | 1 |  |  |  |  |  |  |  |  |  |  |  | 1 |
| AAAC/GTTT | 2 |  |  |  |  |  |  |  |  |  |  |  |  | 2 |
| AAAG/CTTT | 2 |  |  |  |  |  |  |  |  |  |  |  |  | 2 |
| AAAT/ATTT | 5 |  |  |  |  |  |  |  |  |  |  |  |  | 5 |
| AGAT/ATCT | 1 |  |  |  |  |  |  |  |  |  |  |  |  | 1 |
| AATAG/ATTCT | 1 |  |  |  |  |  |  |  |  |  |  |  |  | 1 |

**Table S2.** SSRs identified in the cp genome of *Stephania japonica.*

| Repeats | 3 | 4 | 5 | 6 | 7 | 8 | 9 | 10 | 11 | 12 | 13 | 14 | 15 | 16 | 17 | Total |
| --- | --- | --- | --- | --- | --- | --- | --- | --- | --- | --- | --- | --- | --- | --- | --- | --- |
| A/T | - | - | - | - | - | - | - | 26 | 9 |  | 3 | 4 |  |  | 1 | 43 |
| C/G | - | - | - | - | - | - | - | 1 | 1 |  |  |  |  |  |  | 2 |
| AC/GT | - | - | 1 |  |  |  |  |  |  |  |  |  |  |  |  | 1 |
| AG/CT | - | - | 3 |  |  |  |  |  |  |  |  |  |  |  |  | 3 |
| AT/AT | - | - | 3 | 5 | 2 | 3 |  | 1 |  |  |  |  |  |  |  | 14 |
| AAT/ATT | - | 6 |  |  |  |  |  |  |  |  |  |  |  |  |  | 6 |
| AAAG/CTTT | 2 |  |  |  |  |  |  |  |  |  |  |  |  |  |  | 2 |
| AAAT/ATTT | 2 | 1 |  |  |  |  |  |  |  |  |  |  |  |  |  | 3 |
| AACC/GGTT | 1 |  |  |  |  |  |  |  |  |  |  |  |  |  |  | 1 |
| ATCC/ATGG | 1 |  |  |  |  |  |  |  |  |  |  |  |  |  |  | 1 |
| AAATC/ATTTG | 1 |  |  |  |  |  |  |  |  |  |  |  |  |  |  | 1 |
| AACAT/ATGTT | 1 |  |  |  |  |  |  |  |  |  |  |  |  |  |  | 1 |
| AACCC/GGGTT | 1 |  |  |  |  |  |  |  |  |  |  |  |  |  |  | 1 |
| AATCT/AGATT | 1 |  |  |  |  |  |  |  |  |  |  |  |  |  |  | 1 |

**Table S3.** SSRs identified in the cp genome of *Stephania kwangsiensis.*

| Repeats | 3 | 4 | 5 | 6 | 7 | 8 | 9 | 10 | 11 | 12 | 13 | 14 | 15 | 16 | 17 | 18 | Total |
| --- | --- | --- | --- | --- | --- | --- | --- | --- | --- | --- | --- | --- | --- | --- | --- | --- | --- |
| A/T | - | - | - | - | - | - | - | 18 | 15 | 11 | 4 | 1 |  | 1 |  | 2 | 52 |
| C/G | - | - | - | - | - | - | - | 1 |  |  |  |  |  |  | 1 |  | 2 |
| AC/GT | - | - | 1 |  |  |  |  |  |  |  |  |  |  |  |  |  | 1 |
| AG/CT | - | - | 2 |  |  |  |  |  |  |  |  |  |  |  |  |  | 2 |
| AT/AT | - | - | 1 | 3 | 2 |  | 1 |  |  |  |  |  |  |  |  |  | 7 |
| AAG/CTT | - | 1 |  |  |  |  |  |  |  |  |  |  |  |  |  |  | 1 |
| AAT/ATT | - | 6 | 1 |  |  |  |  |  |  |  |  |  |  |  |  |  | 7 |
| AAAG/CTTT | 1 |  |  |  |  |  |  |  |  |  |  |  |  |  |  |  | 1 |
| AACC/GGTT | 1 |  |  |  |  |  |  |  |  |  |  |  |  |  |  |  | 1 |
| AATT/AATT | 1 |  |  |  |  |  |  |  |  |  |  |  |  |  |  |  | 1 |
| AGAT/ATCT | 1 |  |  |  |  |  |  |  |  |  |  |  |  |  |  |  | 1 |
| ATCC/ATGG | 1 |  |  |  |  |  |  |  |  |  |  |  |  |  |  |  | 1 |
| AACAT/ATGTT | 1 |  |  |  |  |  |  |  |  |  |  |  |  |  |  |  | 1 |
